# Supplementary material for: A facultative plasminogen-independent thrombolytic enzyme from Sipunculus nudus
Source: Nat Commun. 2025 Apr 24;16:3852. doi: 10.1038/s41467-025-58915-y (PMC12022309; doi:10.1038/s41467-025-58915-y)
Supplement: Supplementary file 1 — Supplementary Information [file 41467_2025_58915_MOESM1_ESM.pdf]

Supplementary Information

Including

Supplementary Figures 1-16

Supplementary Tables 1-23

Supplementary Methods

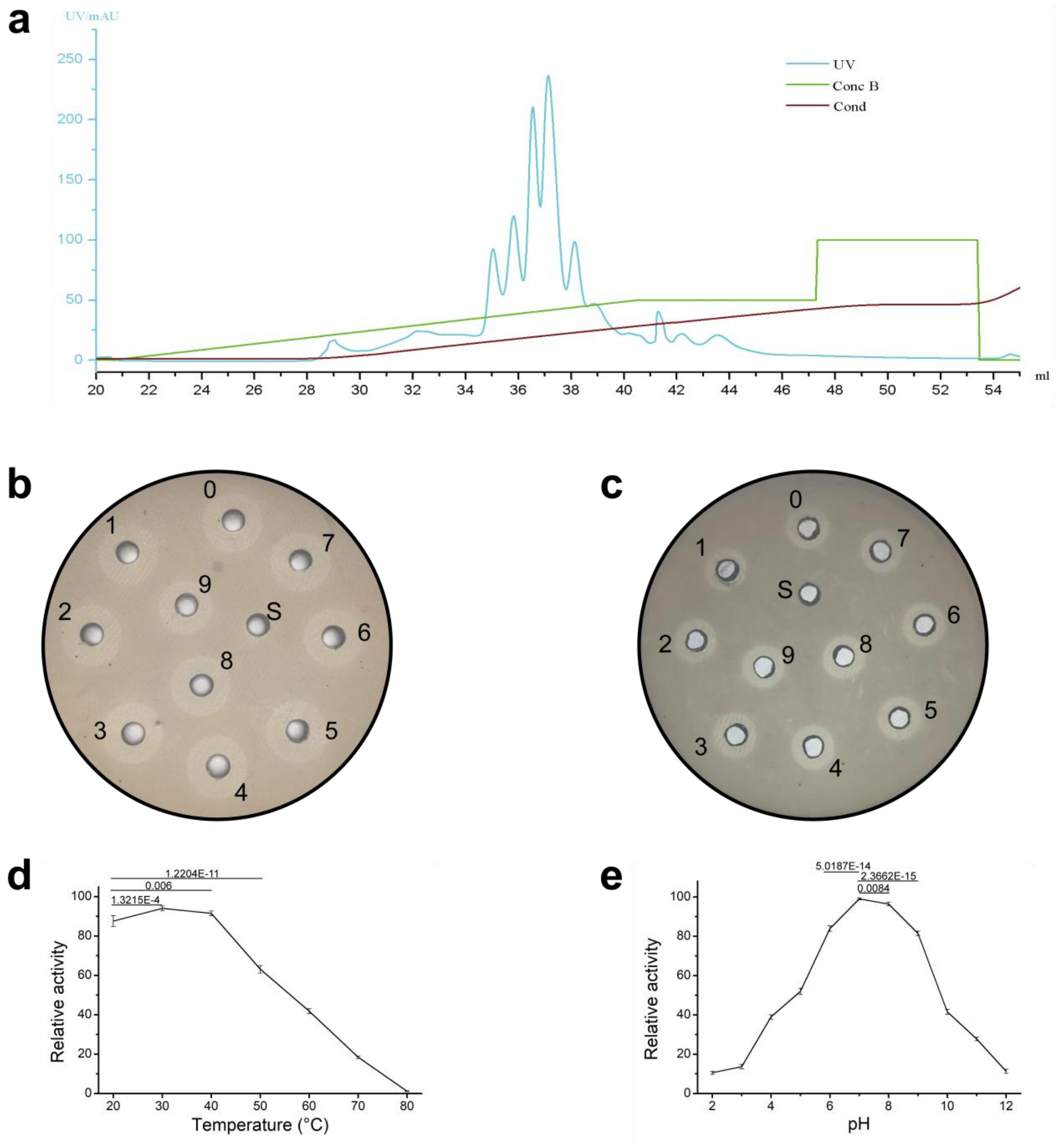

Supplementary Fig. 1 | Enzymatic characterization of snFPITE. a Purification of snFPITE with Resource Q column. **b** Fibrinlytic activity of long-term incubated snFPITE on plasminogen-rich fibrin plate. S=saline, 0=day 0, 1=day 10, 2=day 20, 3=day 30, 4=day 40, 5=day 50, 6=day 60, 7=day 70, 8=day 80, 9=day 90. snFPITE: 1 µg/well. **c** Fibrinlytic activity of long-term incubated snFPITE on plasminogen-free fibrin plate. S=saline, 0=day 0, 1=day 10, 2=day 20, 3=day 30, 4=day 40, 5=day 50, 6=day 60, 7=day 70, 8=day 80, 9=day 90. snFPITE: 1 µg/well. **d** Fibrinlytic activity of snFPPITE under different temperature treatment. Activity values are relative to the

nontreated snFPITE. n = 3 independent experiments. **e** Fibrinlytic activity of snFPPITE under different pH treatment. Activity values were relative to the nontreated snFPITE. n = 3 independent experiments. Five independent experiments were repeated with simimilar results (**b**, **c**). Significance was calculated by repeated-measures one-way ANOVA followed by Tukey and LSD's multiple comparisons test (**d**, **e**). Data are presented as the mean ±SD (**d**, **e**). Source data are provided as a Source Data file.

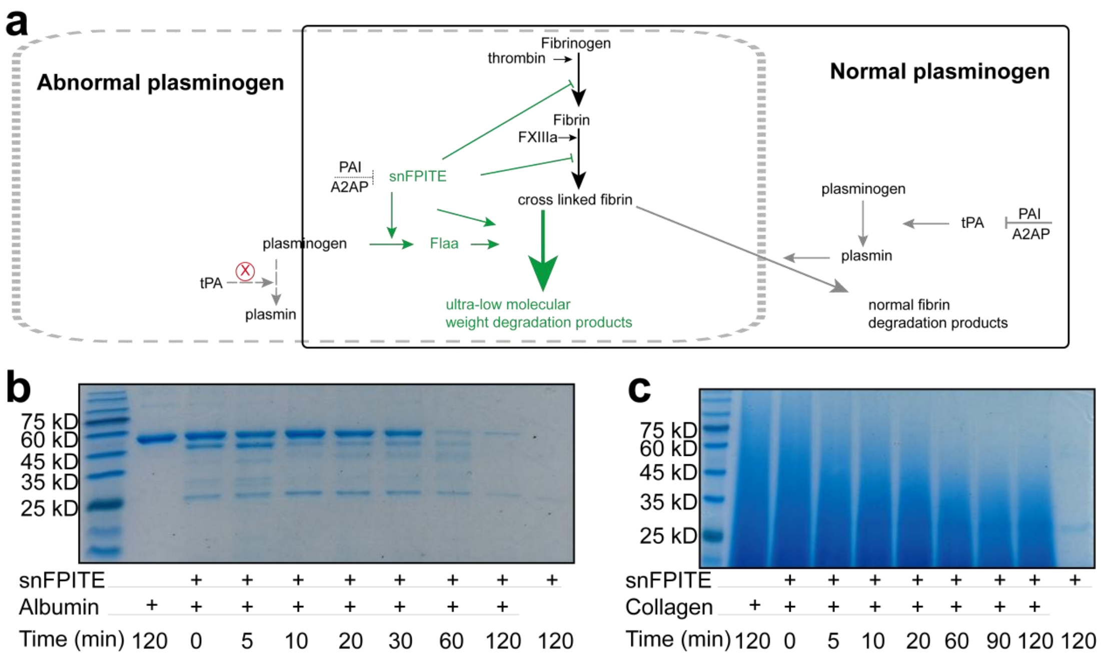

Supplementary Fig. 2 | snFPITE mediated thrombolysis. a snFPITE does not require plaminogen for thrombolysis but lyses better at its presence. Dashed circle indicates snFPITE functioning under an abnormal plasminogen state. i.e., the endogenous plasminogen is under-expressed, mutated, or has a modified conformation, which limits formation of the plasminogen–tPA–fibrin complex. Solid circle indiates under the normal plasminogen state. i.e., the endogenous plasminogen is expressed normaly, with not mutation and conformation change. **b** snFPITE degrades albumin. snFPITE: 0.5 µg/well. albumin: 2 µg/well. **c** snFPITE degrades collagen. snFPITE: 0.5 µg/well. collagen: 2 µg/well. Five independent experiments were repeated with simimilar results (**b**, **c**). Source data are provided as a Source Data file.

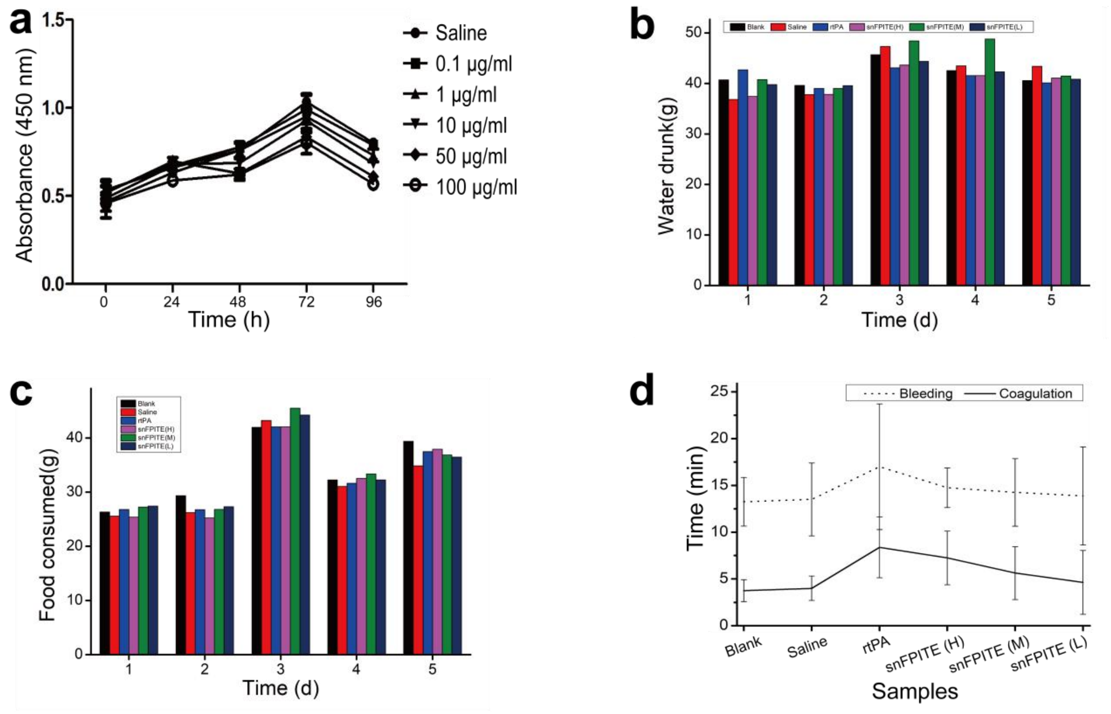

Supplementary Fig. 3 | Safety analysis of snFPITE. a Cell toxicity of snFPITE. Cell viability was shown by the absorbance of CCK8 reagent itself at 450 nm. Saline was set as the control group. *n* = 6 independent experiments. **b** Effects of snFPITE on the water drunk of mouse. rtPA: 0.9 mg/kg, snFPITE (H): 0.9 mg/kg, snFPITE (M): 0.45 mg/kg, snFPITE (L): 0.23 mg/kg. Data are presented as the total weight. *n* = 16 mice per group. **c** Effects of snFPITE on the food consumed of mouse. rtPA: 0.9 mg/kg, snFPITE (H): 0.9 mg/kg, snFPITE (M): 0.45 mg/kg, snFPITE (L): 0.23 mg/kg. Data are presented as the total weight. *n* = 16 mice per group. **d** Effects of snFPITE on bleeding and coagulation system. rtPA: 0.9 mg/kg, snFPITE (H): 0.9 mg/kg, snFPITE (M): 0.45 mg/kg, snFPITE (L): 0.23 mg/kg. *n* = 8 independent experiments. Data are presented as the mean ± SD (**a**, **d**). Significance was calculated by repeated-measures one-way ANOVA followed by Tukey and LSD's multiple comparisons test (**a**, **d**). Source data are provided as a Source Data file.

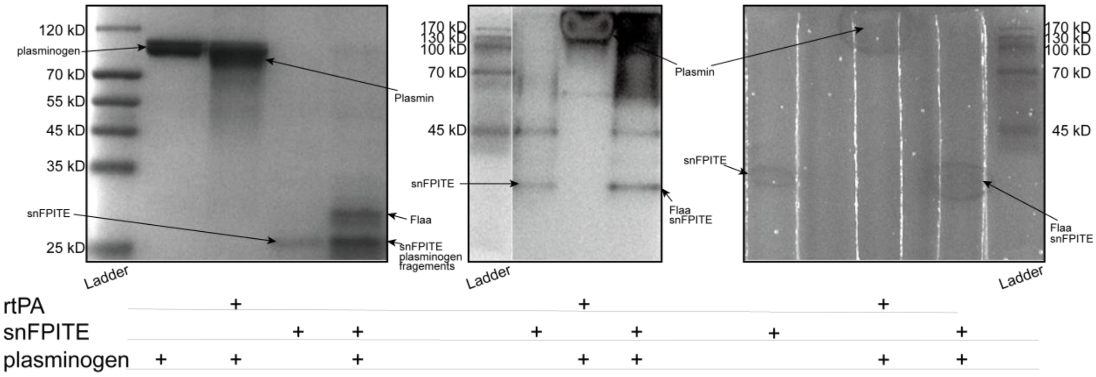

Supplementary Fig. 4 | snFPITE activates plasminogen into Flaa. SDS-PAGE analysis of snFPITE-mediated plasminogen activation (left). rtPA and snFPITE: 0.1 μg/well, plasminogen: 2 μg/well. Native-PAGE analysis of snFPITE-mediated plasminogen activation (middle). rtPA and snFPITE: 0.5 μg/well, plasminogen: 10 μg/well. Native-PAGE based plasminogen-free fibrin plate analysis of snFPITE-mediated plasminogen activation (right). rtPA and snFPITE: 0.5 μg/well, plasminogen: 10 μg/well. Five independent experiments were repeated with simimilar results. Source data are provided as a Source Data file.

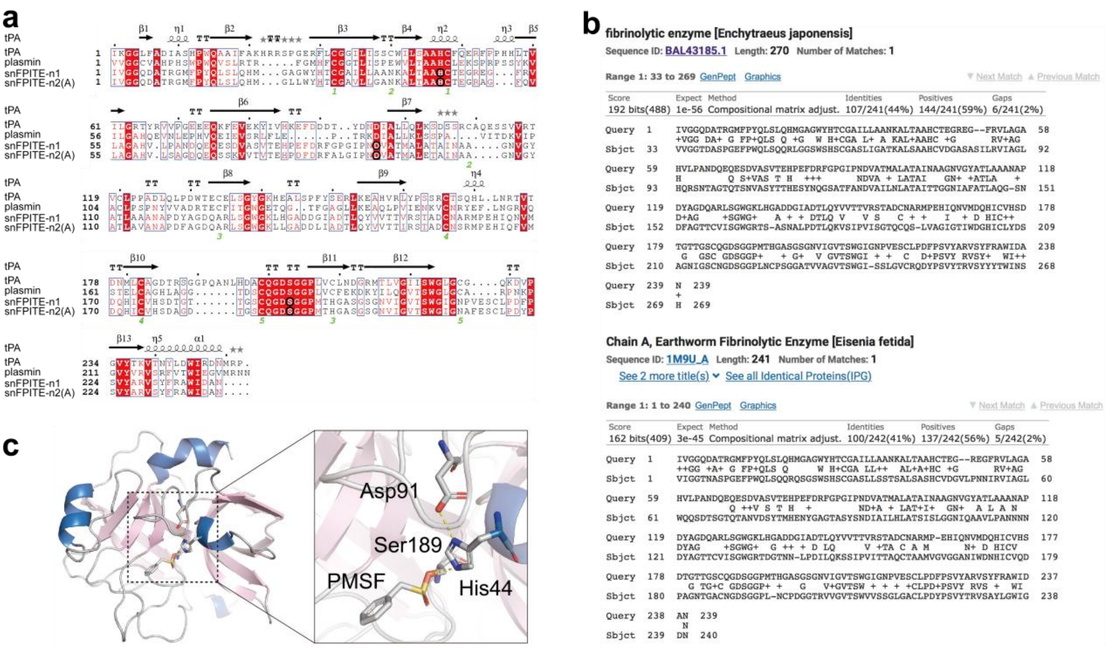

Supplementary Fig. 5 | Sequence and structure analysis of snFPITE crystal. a Sequence conservation analysis between tPA, plasmin, snFPITE-n1 and snFPITE-n2-A. A indicates the subunit A of snFPITE-n2 crystal. Black circles indicate the catalytic amino acids Asp, His, and Ser. **b** Protein sequence alignment of snFPITE-n1 with other representative fibrinolytic enzymes. snFPITE-n1 alignment with fibrinolytic enzyme form *Enchytraeus japonensis* (top) and earthworm fibrinolytic enzyme component A from *Eisenia fetida* (bottom). **c** Crystal structure of snFPITE-n1. Dashed square indicates the catalytic triads of the snFPITE-n1.

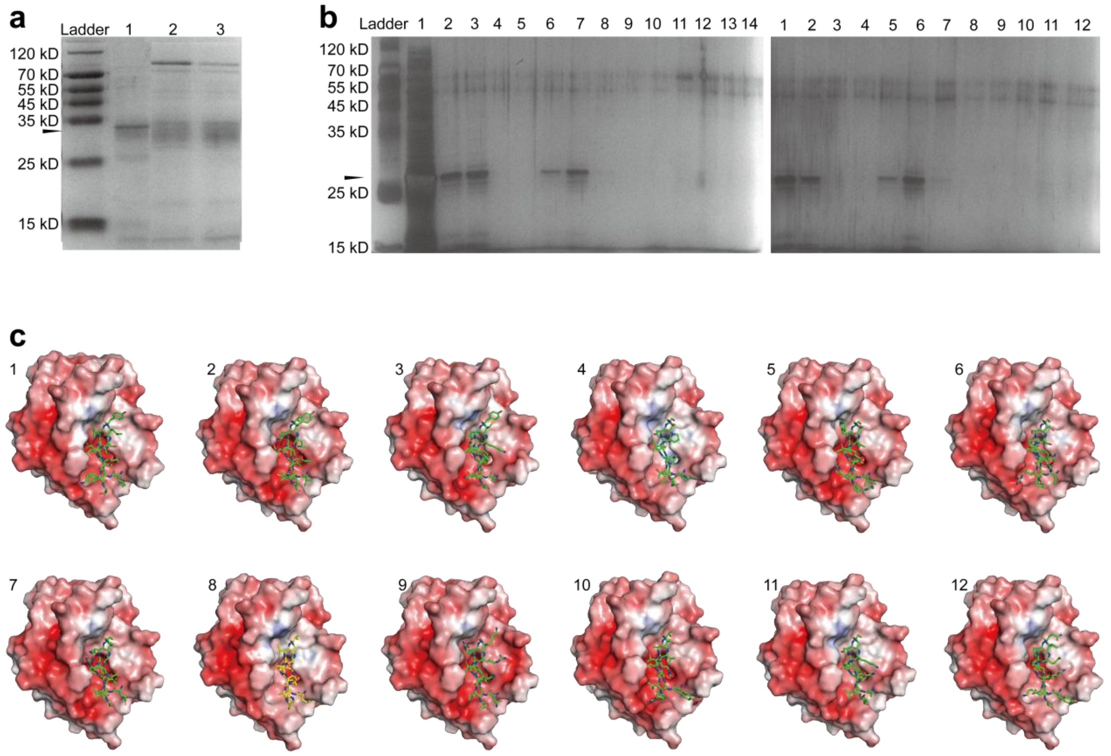

Supplementary Fig. 6 | Regulation and models of snFPITE's plasminogen activation. a Plasminogen activation activity of snFPITE was inhibited by Lysine and Arginine. snFPITE + plasminogen (lane 1); snFPITE + plasminogen + Arginine (lane 2); snFPITE + plasminogen + Lysine (lane 3). snFPITE: 0.1 μg/well, plasminogen: 2 μg/well, Lysine and Arginine: 2.5 mM. Arrow indicates the Flaa fragments. **b**, Affinity purification of snFPITE by Lysine-Sepharose 4B (left) or Arginine-Sepharose 4B (right). The crude snFPITE (lane 1), Tris-HCl breakthrough peak (lanes 2 to 5), 0.15 M NaCl elution peak (lanes 6 to 9), 0.25 M NaCl elution peak (lanes 10 to 11), and the 2 M NaCl + 0.1M NaOH elution peak (lanes 12 to 14). **c** The models of snFPITE binding to the observed twelve major cleavage sites of plasminogen. The peptide fragments LFEKKVYLSECKT (1), VLFEKKVYLSECK (2), SQVRWEYCKIPSC (3), KIPSCDSSPVSTE (4), ITCQKWSSTSPHR (5), TCQKWSSTSPHRP (6), QVEPKKCPGRVVG (7), KCPGRVVGGCVAH (8),

NGKGYRGKRTTVT (9), YRGKRATTVTGTP (10), AAPSFDCGKPQVE (11), and FDCGKPQVEPKKC (12) corresponding to the twelve major cleavage sites of plasminogen were in complex with snFPITE-n1. Five independent experiments were repeated with simimilar results (**a**, **b**). Source data are provided as a Source Data file.

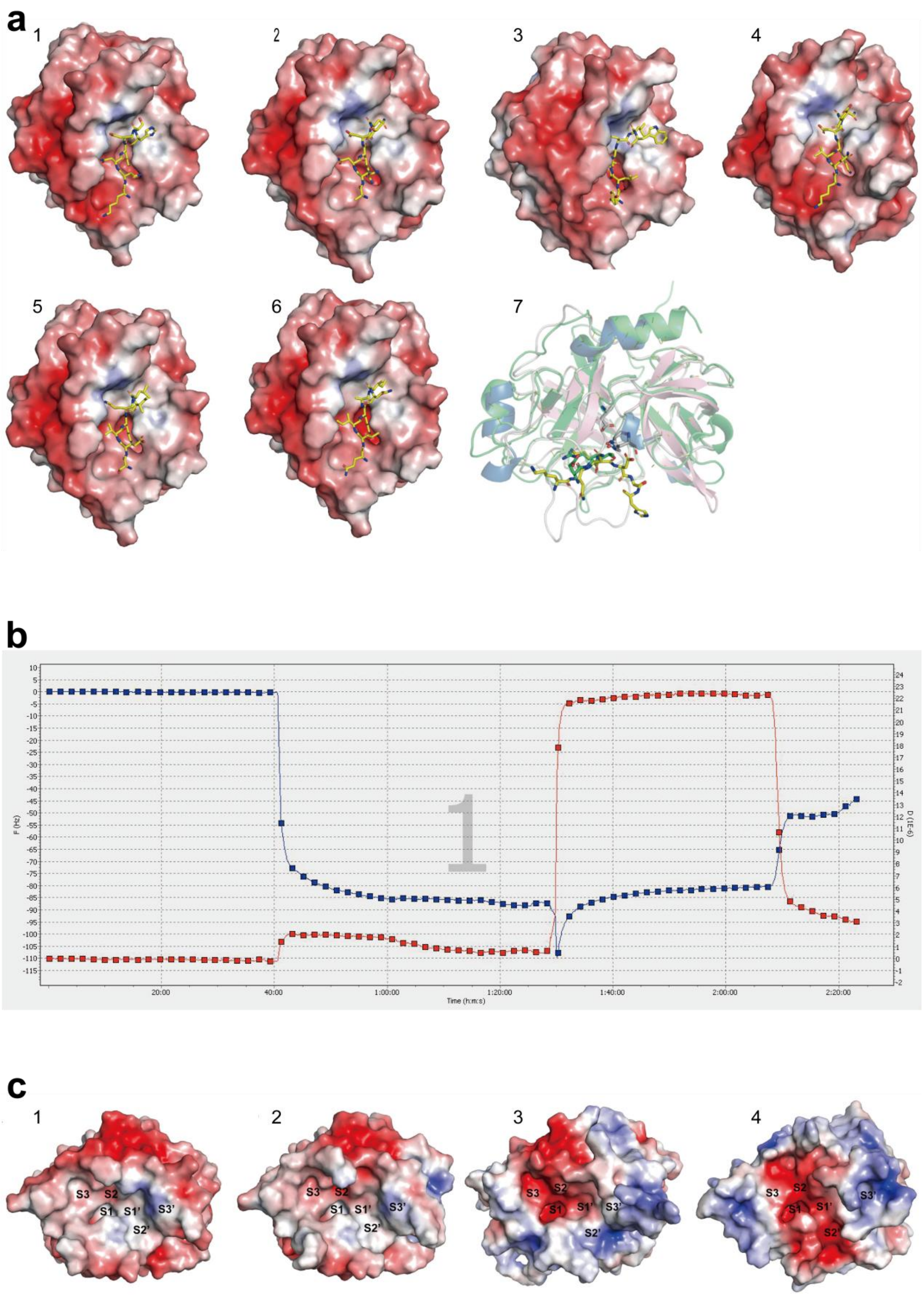

Supplementary Fig. 7 | Models of snFPITE's fibrino(gen)lytic activity. a snFPITE-n1 accommodates peptide fragments from the fibrinogen chains. snFPITE-n1 superimposed with plasmin in complex with peptide KNNKDSH (1) and

ANNRDNT (2) from fibrinogen α chain, YLLKDLW (3) and KQVKDNE (4) from fibrinogen β chain, SEVKQLI (5) and QLIKAIQ (6) fibrinogen γ chain. snFPITE-n1 was superimposed with plasmin in complex with a peptide inhibitor of plasmin (7). **b** The molecular binding performance of snFPITE to fibrinogen on the Quartz crystal microbalance system. The shift up of frequency (blue dot line) and the shift down of the conductivity (red dot line) indicate the direct molecular interaction between snFPITE and fibrinogen. Three independent experiments were repeated with simimilar results. **c** Surface electrostatic potential analysis of the snFPITE pockets. Surface electrostatic potential analysis of the six pockets of snFPITE-n1 (1). Surface electrostatic potential analysis of the six pockets of snFPITE-n2 (molecule A) (2). Surface electrostatic potential analysis of the six pockets of tPA (3). Surface electrostatic potential analysis of the six pockets of plasmin (4). The pockets of snFPITE-n1 and snFPITE-n2 are obvious less positive charged (red) and negative charged (blue) than those of tPA and plasmin.

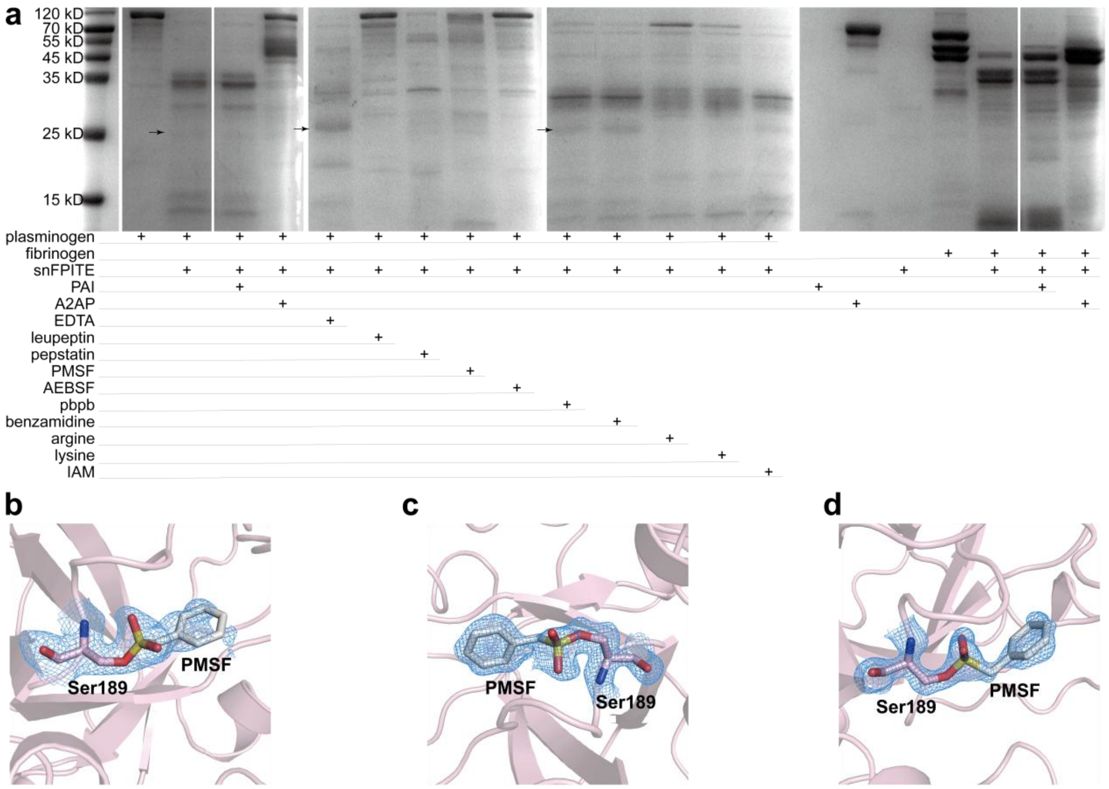

Supplementary Fig. 8 | Inhibitors of snFPITE. a The inhibition of snFPITE's activities. snFPITE: 0.1 μg/well, inhibitors: 2.5 mM. Arrows indicate the Flaa. Three independent experiments were repeated with simimilar results. **b-d** Simulated annealing composite omit maps for Ser189-PMSF in snFPITE-n1 (**b**), snFPITE-n2-A (**c**) and snFPITE-n2-B (**d**) contoured at 0.8 σ, 1.0 σ and 0.9 σ respectively. The protein is shown as pink cartoon. Ser189 is shown as stick with carbon atoms in pink and PMSF is shown as stick with carbon atoms in grey. Nitrogen atom is blue, oxygen atom is red and sulfur atom is yellow. The maps are shown as blue mesh. Source data are provided as a Source Data file.

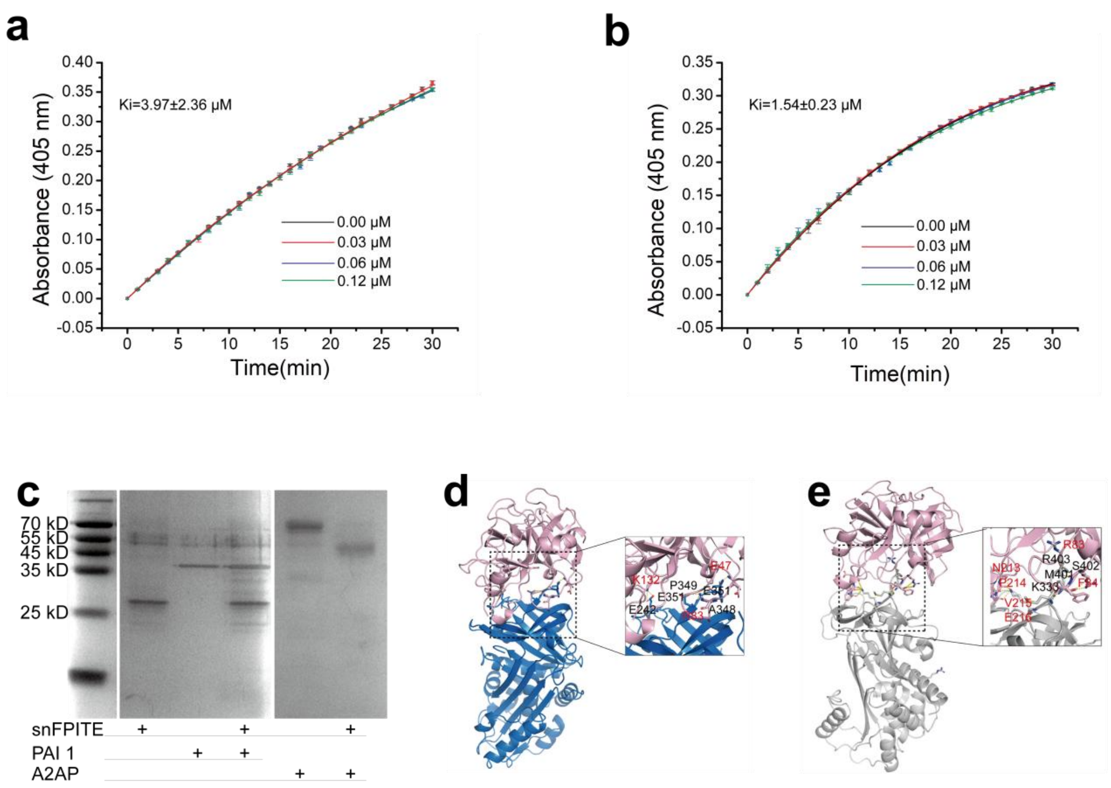

Supplementary Fig. 9 | Inhibition analysis of PAI 1 and A2AP on snFPITE. a K_i analysis of PAI 1 on snFPITE's fibrinolytic activity. n = 6 independent experiments. **b** K_i analysis of PAI1 on snFPITE's plasminogen activation activity. n = 6 independent experiments. **c** snFPITE does not covalent bond to PAI1 (left), but cleaves A2AP (right). snFPITE and inhibitors: 1 μg/well. Five independent experiments were repeated with simimilar results. **d** Model of snFPITE-n1 in complex with human PAI1. There are six hydrogen bonds between snFPITE-n1 and PAI1 which could block the way that plasminogen substrate bound to snFPITE-n1. **e** Model of snFPITE-n1 in complex with human A2AP. There are eight hydrogen bonds at the interface between snFPITE-n1 and A2AP (dashed lines). snFPITE-n1 is shown as a pink cartoon. Human PAI1 is shown as a blue cartoon while human A2AP is shown as a gray cartoon. Hydrogen bonds are shown as yellow dashes. Data are presented as the mean ± SD (**a**, **b**). K_i was calculated by nonlinear regression according to the

"Substrate Inhibition Model" using Origin 8.5 (**a**, **b**). Source data are provided as a Source Data file.

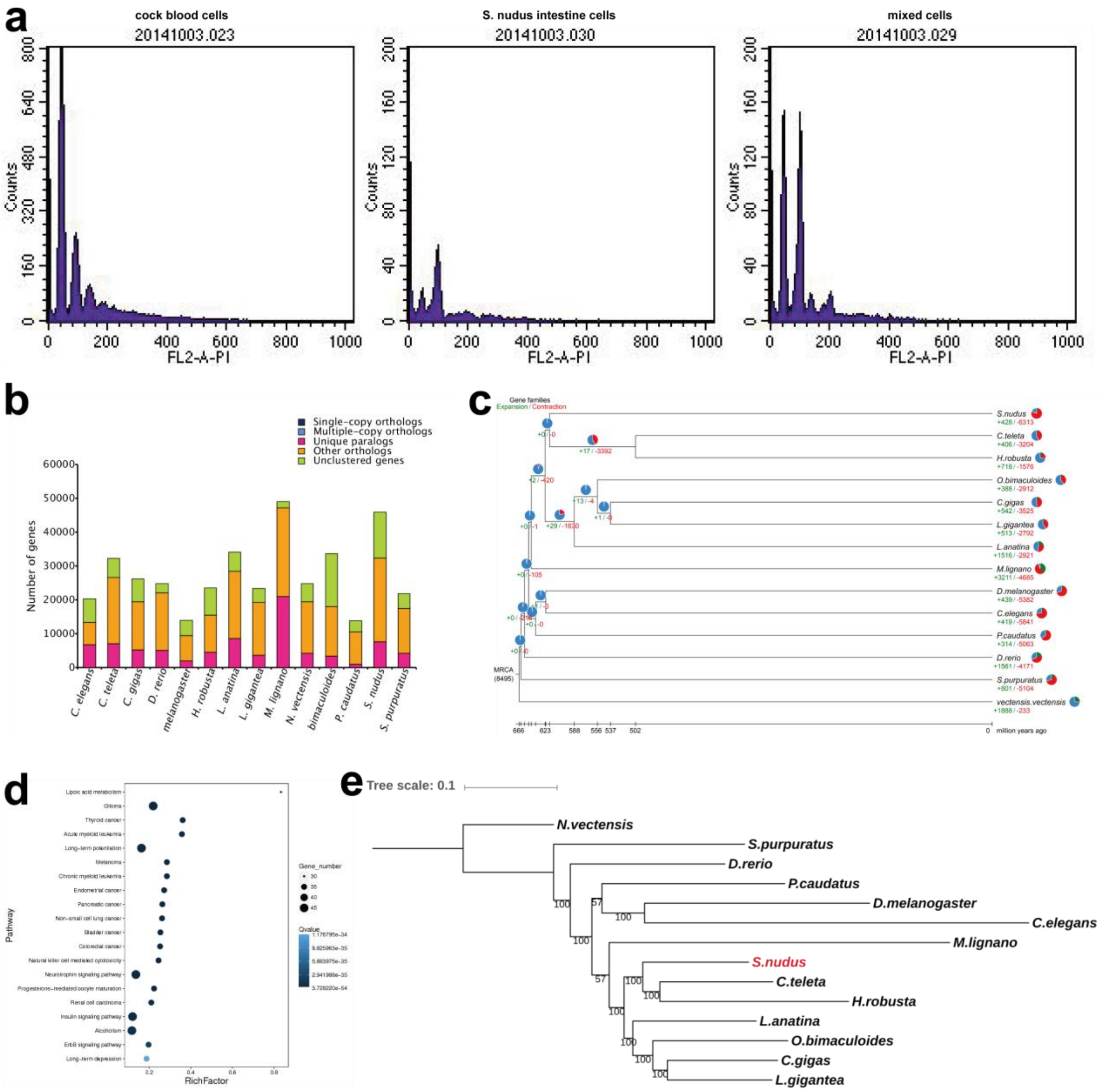

Supplementary Fig. 10 | Features of *S. nudus* genome. a Typical flow cytometry histograms for nuclei isolated from chicken blood cells (left), *S. nudus* intestinal cells (middle), and mixed cells (right). **b** Gene family clusters of the 14 species examined. **c** Expansion and contraction of gene families. Gene family expansions are marked in green, and gene family contractions are represented in red. Their corresponding proportions among the total changes are presented as pie charts using the same colors. Blue portions of the pie charts represent conserved gene families. **d** The significantly enriched KEGG pathway of *S. nudus* genes in significant expansion gene families. Only the statistics for the top 20 pathways are provided. *Q* values represent the

significance of enrichment. Circles indicate the target genes, and the size is proportional to the number of genes. **e** A phylogenetic tree of *S. nudus* and other 13 representative species.

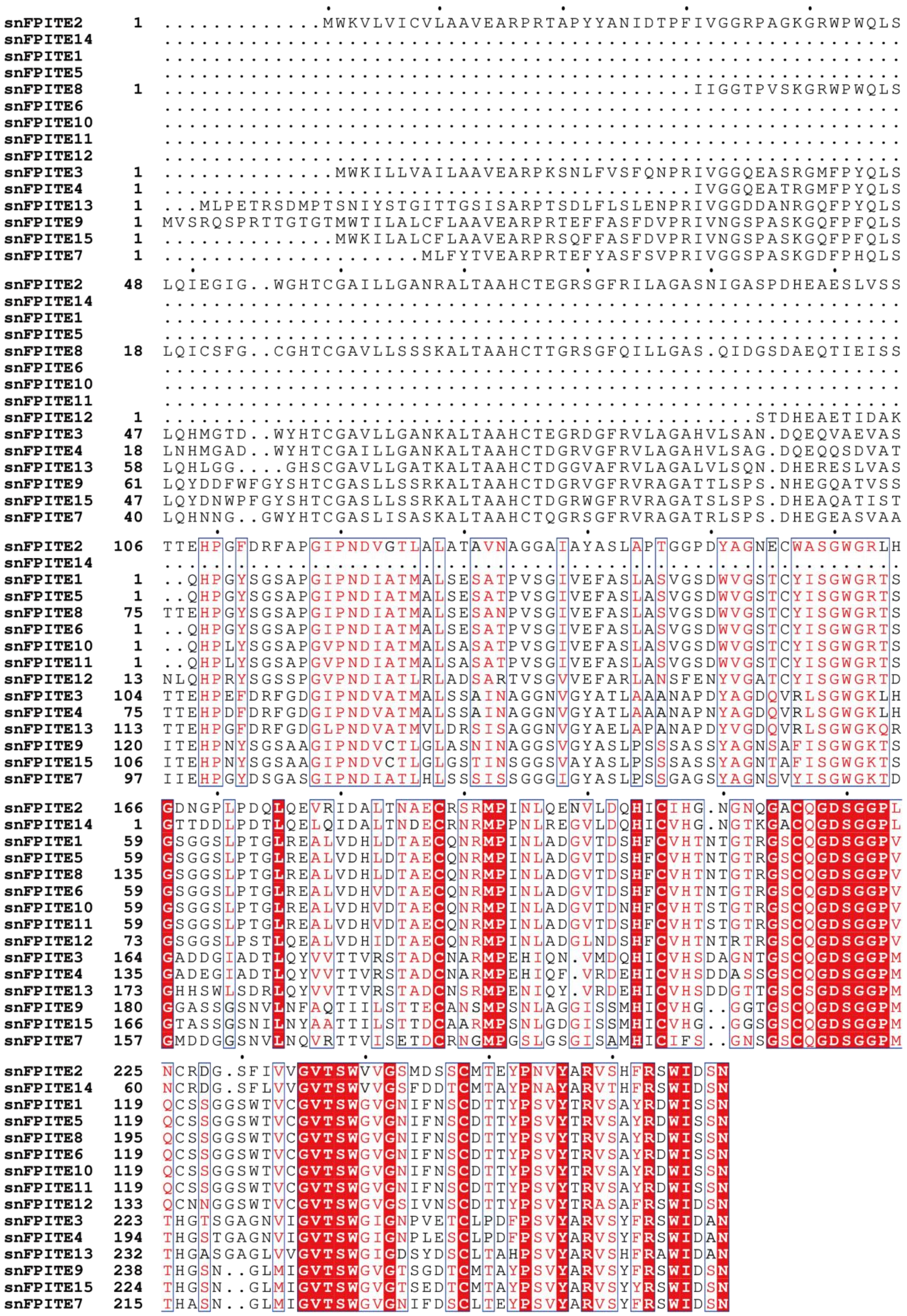

Supplementary Fig. 11 | Protein sequence alignment of the 15 relatively complete *snFPITE* genes.

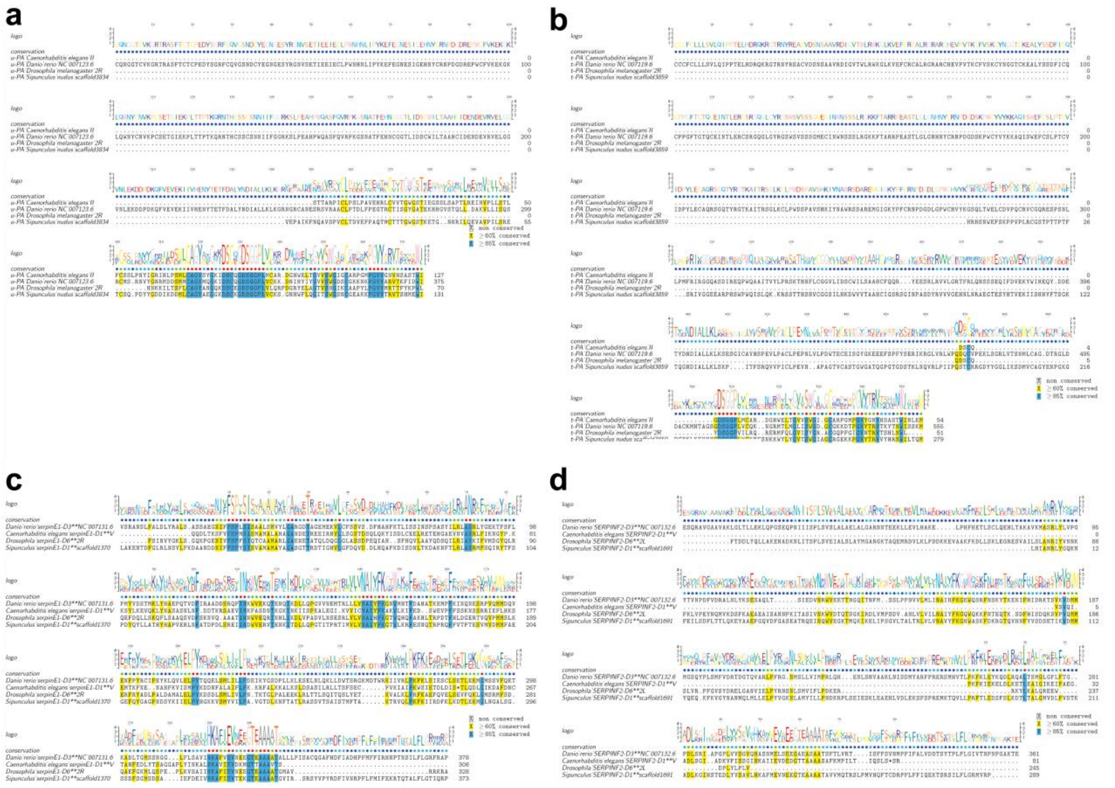

Supplementary Fig. 12 | Conservation comparison of the *uPA*, *tPA*, *PAI1* and *A2AP* among various animals. a *uPA* genes comparison. **b** *tPA* genes comparison. **c** *PAI1* genes comparison. **d** *A2AP* genes comparison. These representative animals include human (*Homo sapiens*), worm (*Caenorhabditis elegans*), fruit fly (*Drosophila melanogaster*) and peanut worm (*Sipunculus nudus*).

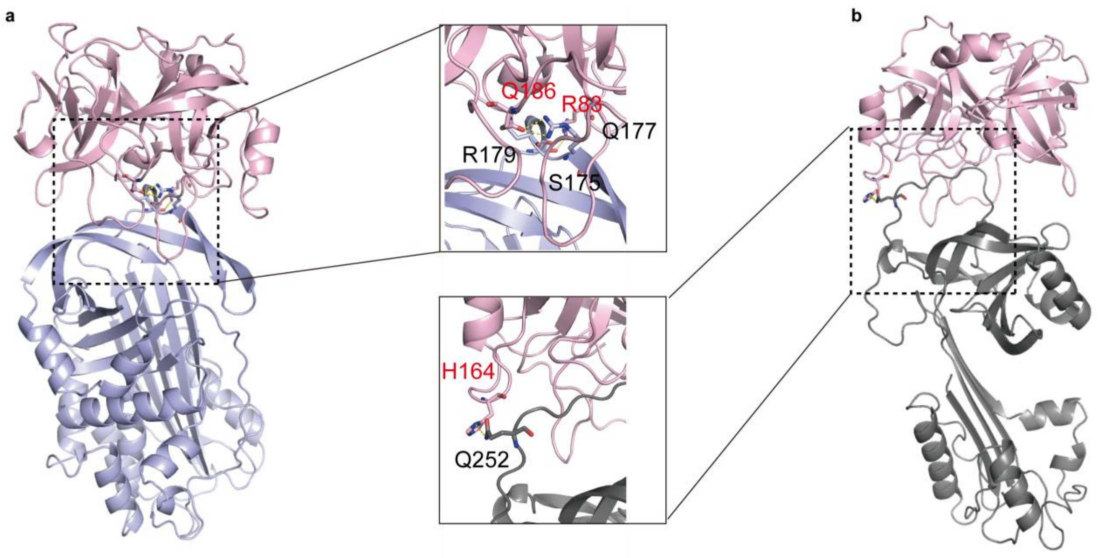

Supplementary Fig. 13 | Models of snFPITE in complex with snPAI or snAP. a Model of snFPITE-n1 in complex with snPAI. **b** Model of snFPITE-n1 in complex with snAP. Structure of snFPITE-n1 is shown as a pink cartoon. Structure of snPAI is shown as a light blue cartoon. Structure of snAP is shown as a dark gray cartoon. Hydrogen bonds are shown as yellow lines.

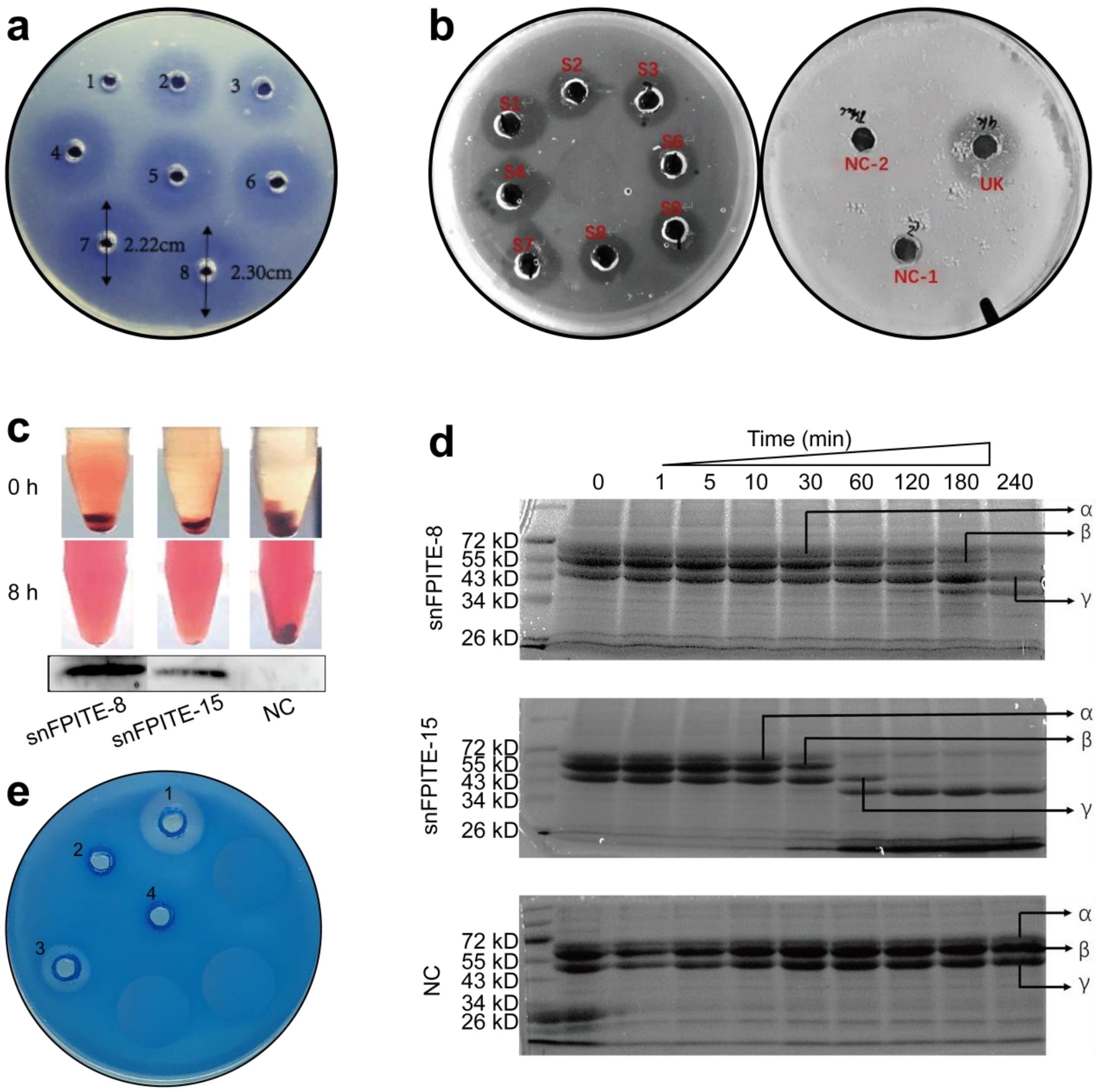

Supplementary Fig. 14 | Fibrinolytic activities of recombinantly expressed snFPITE proteins. a Fibrinolytic acitivity of *snFPITE-c1* expressed in *Pichia Pastoris* system. 0 U/mL UK (#1), 50 U/mL UK (#2), 100 U/mL UK (#3), 200 U/mL UK (#4), 400 U/mL UK (#5), 800 U/mL UK (#6), 1 mg/mL snFPITE-c1 (#7), 1 mg/mL Cygb-snFPITE-c1 (#8). **b** Fibrinolytic activity of 8 *snFPITE* genes through transient transfection in the 293 expression system. Saline (NC-1), culture media from non-transfected group (NC-2), UK (urokinase-type plasminogen activator), culture media from transfected with snFPITE13 (S1), snFPITE4 (S2), snFPITE3 (S3), snFPITE8 (S4), snFPITE7 (S6), snFPITE15 (S7), snFPITE9 (S8) and snFPITE2 (S9)

were added into plasminogen-rich fibrin plate. Their fibrinolytic activities were calculated by their lysing zone. NC-1 and NC-2 were set as negative control. UK was set as positive control. **c** Stable expression and blood clot lysis of 2 *snFPITE* genes. Western blot of 3 snFPITE proteins (bottom). stable expressed 2 snFPITE lyse blood clot (top). NC=saline. **d** Stable expressed 2 snFPITE degrade the α, β and γ chains of fibrinogen. NC=saline. **e** fibrinolytic assay of snFPITE-n1 and microplasmin recombinant expressed in Tn-baculovirus system. 1 mg/mL snFPITE-n1 (#1), 1 mg/mL Tn (#2), 1 mg/mL oPlm (#3), Saline (#4). Three (**c**, **d**) and five (**a**, **b**, **e**) independent experiments were repeated with simimilar results. Source data are provided as a Source Data file.

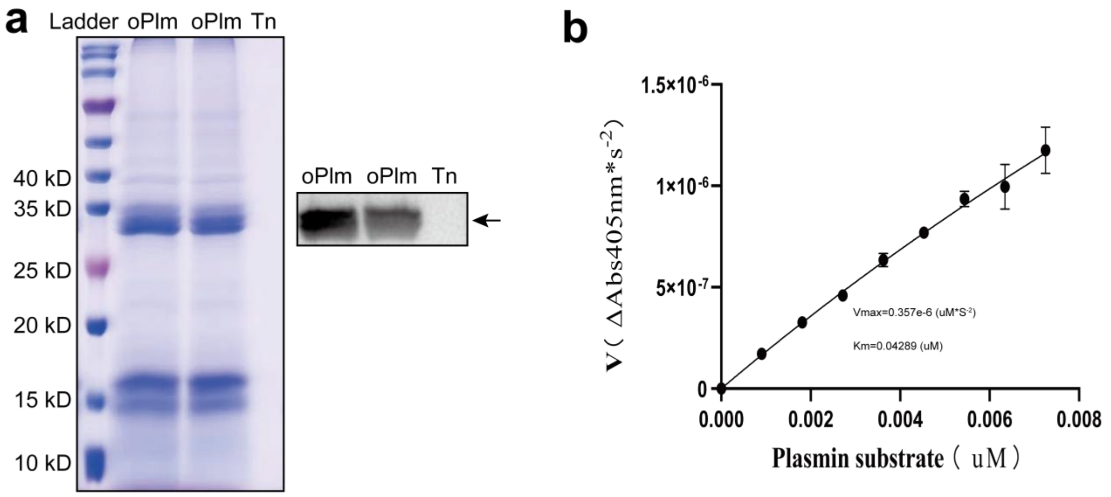

Supplementary Fig. 15 | Fibrinolytic activities of recombinantly expressed oPlm proteins. a SDS-PAGE and Western blot analysis of oPlm. Arrow indicates the oPlm. Culture media from non-transfected Tn cell was set as negative control. 20 µL/well. **b** Enzyme kinetics of oPlm degrading fibrin. Data were presented as the mean ± SD (n = 6). K_m and V_{max} were calculated by nonlinear regression according to the Michaelis–Mentenequation using GraphPad prim. V_{max} was further calculated by the standard curve of standard agent (y = 0.225e-4 • x). y: ΔAbs405 nm; x: concentration of standard agent (µM). Three independent experiments were repeated with simimilar results (**a**, **b**).Source data are provided as a Source Data file.

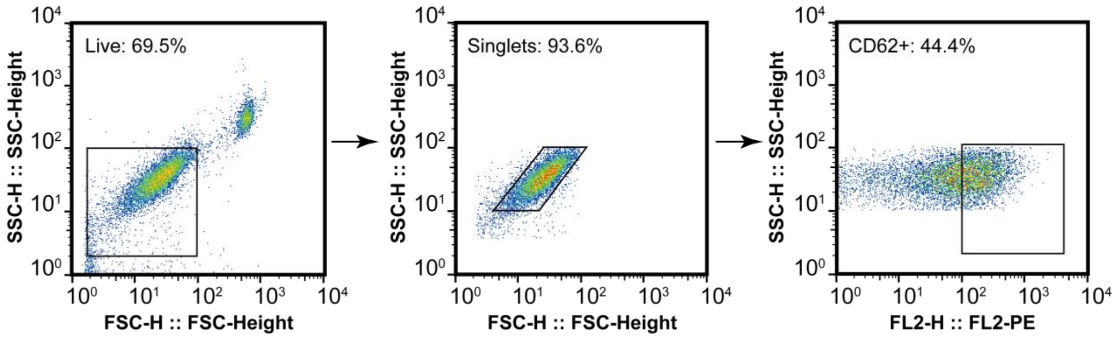

Supplementary Fig. 16 | Flow cytometry gating strategy of Figure 3c.

Supplementary Table 1 | Four coagulation tests of snFPITE in mouse model.

Test items / Values (mg/L) / Samples	Prothrombin time (PT)	Activation part thrombin time (APTT)	Fibrinogen (FIB)	Thrombin time (TT)
Saline-1	8.2	27.2	2.94	13.3
Saline-2	8.1	27.7	3.06	13.1
Saline-3	8.3	27.5	3.11	12.6
Saline-4	7.5	26.8	3.24	13.5
Saline-5	8.4	28.2	2.87	14.2
Saline-6	7.9	27.6	2.65	11.4
rtPA-1	9.3	40.5	2.72	15.7
rtPA-2	9.1	38.6	2.38	13.4
rtPA-3	9.6	39.3	2.66	15.2
rtPA-4	8.6	41.5	2.39	16.4
rtPA-5	10.7	37.6	2.92	14.9
rtPA-6	10.9	38.9	2.44	12.6
snFPITE(H)-1	10.4	41.2	2.25	14.1
snFPITE(H)-2	11.3	39.6	2.14	15.2
snFPITE(H)-3	12.3	40.5	2.01	15.6
snFPITE(H)-4	11.2	43.2	2.34	13.4
snFPITE(H)-5	13.5	42.1	2.54	16.7
snFPITE(H)-6	12.6	42.8	2.17	16.6
snFPITE(M)-1	8.7	33.4	3.03	13.7
snFPITE(M)-2	8.9	30.5	2.75	14.8
snFPITE(M)-3	9.3	35.1	2.88	13.5
snFPITE(M)-4	10.5	29.4	2.63	14.1
snFPITE(M)-5	9.4	28.6	2.32	14.6
snFPITE(M)-6	8.9	31.7	3.21	13.4
snFPITE(L)-1	8.2	28.5	3.16	13.6
snFPITE(L)-2	8.3	26.9	2.85	11.6
snFPITE(L)-3	8.6	27.7	2.88	14.6
snFPITE(L)-4	9.1	28.3	3.21	12.7
snFPITE(L)-5	8.8	27.6	2.98	14.8
snFPITE(L)-6	7.9	29.6	3.27	12.5

Supplementary Table 2 | snFPITE degrades D-dimer in *ex vivo* model.

Blood sample ID \ Value (mg/L) \ Group	Saline	snFPITE
319	3.19	1.32
316	15.17	--
305	250	0
293	4.4	0
289	6.06	0.23

Note: "--" indicated.undetectable.

Supplementary Table 3 | snFPITE degrades serum protein in *ex vivo* model.

Samples \ Test items / Value(g/L)	Total protein (TP)	Albumin (ALB)	Globulin (GLB)
Saline-1	81.7	48.8	31.2
Saline-2	79.3	49.2	29.6
Saline-3	75.9	47.3	28.6
rtPA-1	79.7	48.1	31.6
rtPA-2	78.3	49.3	30.4
rtPA-3	75.1	47.6	28.7
snFPITE(H)-1	56.5	34.1	22.4
snFPITE(H)-2	62.8	37.8	25.0
snFPITE(H)-3	63.7	38.9	27.4

Supplementary Table 4 | snFPITE degrades serum protein in *in vitro* model.

Samples \ Value(g/L) \ Test items	Total protein (TP)	Albumin (ALB)	Globulin (GLB)
Blank-1	44.2	32.6	12.8
Blank-2	44.3	30.4	13.9
Blank-3	43.5	31.2	12.1
Saline-1	44.9	32.7	12.6
Saline-2	44.2	32.6	11.6
Saline-3	44.7	31.1	12.3
rtPA-1	42.3	30.0	12.3
rtPA-2	42.5	31.2	12.8
rtPA-3	43.7	32.7	11.7
snFPITE(H)-1	42.6	29.5	13.1
snFPITE(H)-2	43.6	30.7	12.3
snFPITE(H)-3	42.4	31.2	11.8
snFPITE(M)-1	41.5	29.1	12.4
snFPITE(M)-2	46.7	35.2	11.5
snFPITE(M)-3	43.6	32.1	12.1
snFPITE(L)-1	41.3	29	12.3
snFPITE(L)-2	45.8	33.7	12.1
snFPITE(L)-3	42.8	30.4	13.2

Supplementary Table 5 | Trypsin and chymotrypsin activities of snFPITE.

Test items / Activity (U/mg) / Samples	Tryspin	Chymotrypsin
1	537.2	0.12
2	564.1	0.25
3	511.6	0.17
4	573.5	0.31
5	500.4	0.08
6	545.9	0.21

Supplementary Table 6 | Blood concentration of Cy7 conjugated snFPITE.

Samples / Absorbance (746 nm) / Time points (min)	0	10	30	60	120	240	480
1	0.0661	0.1027	0.0761	0.0856	0.0839	0.0865	0.0760
2	0.0650	0.1074	0.0768	0.0878	0.0843	0.0913	0.0764
3	0.0649	0.1045	0.0782	0.0907	0.0850	0.0860	0.0778
4	0.0665	0.1039	0.0767	0.0865	0.0848	0.0874	0.0768
5	0.0648	0.1098	0.0777	0.0891	0.0847	0.0914	0.0768
6	0.0652	0.1054	0.0785	0.0929	0.0861	0.0863	0.0782
7	0.0670	0.1050	0.0771	0.0872	0.0860	0.0882	0.0768
8	0.0648	0.1106	0.0781	0.0895	0.0854	0.0914	0.0772
9	0.0654	0.1062	0.0786	0.0930	0.0860	0.0864	0.0788

Supplementary Table 7 | Blood routine parameters test.

Test items / Values / Samples	RBC (e+12/L)	HGB(g/L)	HCT (%)	MCV(fl)	WBC(e+9/L)	LYM(e+9/L)	Neu #(e+9/L)	PLT (e+9/L)	MPV(fl)	P-LCR(%)	PCT (%)
Saline-1	9.33	139	48.9	52.4	1.9	1.85	0.05	876	6.8	5.3	0.6
Saline-2	9.21	140	48.3	52.4	1.76	1.7	0.06	871	6.8	5	0.59
Saline-3	8.92	133	48.1	51.7	2.16	2.11	0.09	784	6.3	4.8	0.51
rtPA-1	7.78	120	41.7	53.6	1.16	1.01	0.11	450	7	5.3	0.31
rtPA-2	8.37	129	44.8	53.5	2.86	2.61	0.18	545	6.8	4.7	0.24
rtPA-3	8.51	118	45.3	51.6	1.67	1.82	0.13	630	6.3	5.1	0.33
snFPITE(H)-1	7.29	115	37.9	52	2.47	2.31	0.12	534	6.6	3.4	0.22
snFPITE(H)-2	7.11	114	36.9	51.9	2.55	2.38	0.12	590	6.6	3.6	0.39
snFPITE(H)-3	7.68	123	38.2	50.1	2.34	2.11	0.08	710	6.8	3.9	0.51
snFPITE(M)-1	7.67	122	41.9	54.6	2.36	3.07	0.25	582	6.4	2.5	0.69
snFPITE(M)-2	7.69	124	41.8	54.4	2.34	2.54	0.3	623	6.8	5.5	0.76
snFPITE(M)-3	7.88	135	43.6	52.1	2.35	2.88	0.21	760	6.1	6.2	0.77
snFPITE(L)-1	8.41	132	45.3	53.9	2.77	3.29	0.13	608	6.6	3.4	0.14
snFPITE(L)-2	8.34	135	44.8	53.7	2.66	2.22	0.13	793	7.1	8.8	0.14
snFPITE(L)-3	8.63	142	42.6	51.3	2.64	2.25	0.10	652	6.3	5.2	0.78

Supplementary Table 8 | Cleavage sites involved in the process of snFPITE mediated plaminogen activation.

Steps<br>Sites	Major sites	Minor sites
Step one	97K/98V, 344W/345E	96K/97K, 99Y/100L, 104K/105T, 111Y/112R, 343R/344W, 346Y/347C, 348K/349I, 352C/353D, 359T/360E
Step two	127W/128S, 575K/576K	120N/121G, 126K/127W, 580R/581V
Step three	489Y/490R	479E/480D, 492K/493R, 493R/494A
Step four	565F/566D	569K/570P

Note: Sites were the cleavage sites on the plasminogen (AAA60113.1); Steps were the mainly steps involved in the plasminogen activation.

Supplementary Table 9 | Data collection and refinement statistics (molecular replacement).

	snFPITE-n1	snFPITE-n2
**Data collection**		
Space group	P 21 2 21	P 21 21 21
Cell dimensions		
a, *b*, *c* (Å)	40.315, 43.73, 121.59	39.262, 59.738, 193.702
α, β, γ (°)	90, 90, 90	90, 90, 90
Resolution (Å)	43.73 - 1.99 (2.04 - 1.99) *	96.85 - 1.50 (1.53 - 1.50)
R_{merge}	0.021 (0.025)	0.077 (0.628)
$I / \sigma I$	24.6 (14.9)	12.6 (2.6)
Completeness (%)	99.2 (95.9)	96.0 (99.9)
Redundancy	3.2 (2.5)	5.7 (5.7)
**Refinement**		
Resolution (Å)	1.99	1.50
No. reflections	15312	71213
R_{work} / R_{free}	0.1530 / 0.2004	0.1633 / 0.1870
No. atoms	2001	4045
Protein	1749	3520
Ligand/ion	15	80
Solvent	237	445
B-factors	23.36	20.28
Protein	22.433	18.57
Ligand/ion	34.18	41.15
Solvent	29.52	30.05
R.m.s. deviations		
Bond lengths (Å)	0.009	0.012
Bond angles (°)	0.73	1.13

*Values in parentheses are for highest-resolution shell.

Supplementary Table 10 | *snFPITE* genes identified from *S. nudus*.

No.	ID	Identification methods	Notes
1	snFPITE-c1	Clone, based on conserved sequences	Functionally verified with Pichia sastoris system
2	snFPITE-c2	Clone, based on the transcriptome sequence	Protein sequence is identical to snFPITE-n1
3	snFPITE-c3	Clone, based on the transcriptome sequence	
4	snFPITE-c4	Clone, based on the transcriptome sequence	
5	snFPITE-c5	Clone, based on the transcriptome sequence	
6	snFPITE-c6	Clone, based on the transcriptome sequence	
7	snFPITE-c7	Clone, based on the transcriptome sequence	
8	snFPITE-c8	Clone, based on the transcriptome sequence	
9	snFPITE-c9	Clone, based on the transcriptome sequence	
10	snFPITE-c10	Clone, based on the transcriptome sequence	
11	snFPITE-n1	Mass spectroscopy	Functionally verified with Baculovirus system
12	snFPITE-n2	Mass spectroscopy	Contain A and B subunit, sequence of B is identical to snFPITE-n1.
13	snFPITE1	Genome sequencing	
14	snFPITE2	Genome sequencing	Functionally verified with HEK293 system
15	snFPITE3	Genome sequencing	Functionally verified with HEK293 system
16	snFPITE4	Genome sequencing	Functionally verified with HEK293 system
17	snFPITE5	Genome sequencing	
18	snFPITE6	Genome sequencing	
19	snFPITE7	Genome sequencing	Functionally verified with HEK293 system
20	snFPITE8	Genome sequencing	Functionally verified with HEK293 system
21	snFPITE9	Genome sequencing	Functionally verified with HEK293 system
22	snFPITE10	Genome sequencing	
23	snFPITE11	Genome sequencing	
24	snFPITE12	Genome sequencing	
25	snFPITE13	Genome sequencing	Functionally verified with HEK293 system
26	snFPITE14	Genome sequencing	
27	snFPITE15	Genome sequencing	Functionally verified with HEK293 system
28	snFPITE-p1	Genome sequencing	

Supplementary Table 11 | Statistics of sequencing data using the Illumina sequencing platform.

Paired-end libraries	Insert size (kb)	Reads length (bp) (Raw / Clean)	Raw data (Gb)	Clean data (Gb)	Sequence depth (×) of clean data
	0.45	250 / 248	181.89	158.75	95.06
	0.8	125 / 123	118.90	107.48	64.36
Solexa	2	49 / 47	65.35	21.21	12.70
Reads	5	49 / 47	66.75	14.50	8.68
	10	49 / 47	60.00	13.86	8.30
	20	49 / 47	30.23	7.45	4.46
	40	49 / 47	28.67	8.25	4.94
Total	/	/	551.81	331.50	198.50

Note: We constructed two short libraries with insert size of 450 bp and 800 bp, respectively. The calculation of sequence depth was based on the assembled genome size of 1.67 Gb.

Supplementary Table 12 | Genome size estimation by the k-mer method with K=31.

K	Total *k*-mer Number	Peak depth	Genome size (Gb)	Used bases (bp)	Used reads
31	127,777,276,942	78	1.64	158,753,478,016	6,401,349,926

Note: The genome size was estimated according to the following formula: Genome size = (Total *k*-mer Number /Peak Depth).

Supplementary Table 13 | Statistics of the PacBio RS sequencing.

Libraries	Insert size (kb)	Cell number	Total subread length (Gb)	Sequence depth (×) *
PacBio Reads	20	43	36.17	21.23

Note: *The calculation of sequence depth was based on the assembled genome size of 1.67 Gb.

Supplementary Table 14 | Statistics of the *S. nudus* genome assembly.

Parameter	Contig		Scaffold	
	Size (bp)	Number	Size (bp)	Number
N90	7,957	47,150	160,856	2,652
N80	15,062	32,816	327,786	1,955
N70	21,880	24,025	441,155	1,504
N60	28,900	17,664	539,685	1,151
N50	36,759	12,733	651,131	858
Longest	477,862	/	3,350,528	/
Total Size	1,604,860,243	/	1,727,737,036	/
Total Number (>=100 bp)	/	136948	/	72,674
Total Number (>=2 kb)	/	72565	/	16,494

Supplementary Table 15 | Statistics of repetitive elements in the *S. nudus* genome.

Catego	RepBase TEs		TE Proteins		De novo		Combined TEs	
	Length (bp)	% of Geno	Length (bp)	% of Geno	Length (bp)	% of Geno	Length (bp)	% of Geno
DNA	35,872,6	2.0763	3,438,28	0.1990	319,700,4	18.504	337,391,2	19.527
LINE	16,896,3	0.9779	26,899,7	1.5569	98,988,48	5.7294	119,072,9	6.8918
SINE	432,076	0.0250	0	0	3,639,846	0.2107	4,023,736	0.2329
LTR	20,314,9	1.1758	22,659,5	1.3115	206,789,2	11.968	215,881,7	12.495
Other	34,630	0.0020	0	0	85,675	0.0050	120,305	0.0070
Unkno	0	0	0	0	42,935,87	2.4851	42,935,87	2.4851
Total	66,108,4	3.8263	52,971,2	3.0659	614,147,5	35.546	641,450,8	37.126

Note: Repeat elements were identified by different methods and then combined into the final repeat set.

Supplementary Table 16 | Gene annotation of the *S. nudus* genome.

Gene set	Number	Average gene length (bp)	Average CDS length (bp)	Average exon per gene	Average exon length (bp)	Average intron length (bp)
C. teleta	33,866	19,937.37	554.32	2.74	202.31	11,140.22
C. gigas	19,459	22,357.16	588.24	3.24	181.29	9,697.84
D. rerio	18,722	40,116.89	634.71	3.21	198.00	17,900.99
O. bimaculoides	27,847	24,794.96	550.73	2.54	216.76	15,735.80
P. caudatus	17,986	22,616.73	552.79	2.90	190.34	11,586.63
RNA-Seq(transcripts)	70,689	12,633.40	1,793.49	5.18	346.54	2,596.09
Iso-Seq	14,085	15,796.82	2,493.06	5.56	448.35	2,917.20
Maker (Final set)	45,896	13,850.23	1,021.62	5.81	269.59	2,548.85

Note: Those genes with previous stop codons or ambiguous bases were filtered in the final gene set.

Supplementary Table 17 | Function annotation of *S. nudus* genes.

**Classification**		**Number**	**Percentage (%)**
Total genes		45,896	/
Annotated	InterPro	33,098	72.12
	GO	20,899	45.54
	COG	11,282	24.58
	KEGG	28,313	61.69
	Swissprot	27,404	59.71
	TrEMBL	34,618	75.43
	Nr	34,743	75.70
All annotated		36,951	80.51

Supplementary Table 18 | Gene families clustered in 14 representative species.

**Species**	**Total genes**	Unclustered genes	**Famili es**	**Unique families**	**Average genes per family**
C. elegans	20,184	6,830	4,983	1,268	2.68
C. teleta	32,173	5,573	13,824	1,410	1.92
C. gigas	26,088	6,701	10,547	1,230	1.84
D. rerio	24,700	2,674	8,306	1,042	2.65
D. melanogaster	13,866	4,466	5,131	521	1.83
H. robusta	23,432	7,928	7,705	802	2.01
L. anatina	34,075	5,636	13,960	2,704	2.04
L. gigantea	23,340	4,092	11,798	771	1.63
M. lignano	49,016	1,930	11,049	5,494	4.26
N. vectensis	24,773	5,310	9,656	1,160	2.02
O. bimaculoides	33,608	15,584	9,988	746	1.8
P. caudatus	13,719	3,154	7,313	271	1.44
S. purpuratus	21,854	4,483	7,989	823	2.17
S. nudus	45,896	13,527	15,607	1,941	2.07

Note: Unique families: the specific gene families of this species.

Unclustered genes: those genes that are not clustered into any known gene family.

Supplementary Table 19 | Significantly enriched KEGG pathway of *S. nudus* genes in unique gene families.

Pathway	Sample (3,440)	Background (28,360)	Q-value	Pathway ID
Tuberculosis	457	1204	9.891591e-121	ko05152
Phagosome	455	1236	8.947428e-115	ko04145
NOD-like receptor signaling pathway	241	662	6.016923e-58	ko04621
Salmonella infection	146	316	1.480982e-49	ko05132
Legionellosis	156	367	3.042418e-47	ko05134
Hematopoietic cell lineage	175	446	3.136179e-47	ko04640
Thyroid hormone signaling pathway	205	808	3.733105e-24	ko04919
MicroRNAs in cancer	240	1,056	2.849148e-21	ko05206
Notch signaling pathway	150	546	3.278668e-21	ko04330
Prion diseases	132	508	1.840520e-16	ko05020
Cell adhesion molecules (CAMs)	111	403	8.054951e-16	ko04514
Epstein-Barr virus infection	175	779	4.986685e-15	ko05169
Dorso-ventral axis formation	132	533	9.304868e-15	ko04320
Malaria	72	232	3.506186e-13	ko05144
Staphylococcus aureus infection	83	309	2.506523e-11	ko05150
ECM-receptor interaction	124	576	1.807470e-09	ko04512
Complement and coagulation cascades	78	359	3.241786e-06	ko04610
Intestinal immune network for IgA production	14	27	1.003254e-05	ko04672
Protein digestion and absorption	124	695	9.379729e-05	ko04974
PI3K-Akt signaling pathway	161	989	8.065024e-04	ko04151
Focal adhesion	148	930	4.324576e-03	ko04510
Transcriptional misregulation in cancer	78	440	4.847655e-03	ko05202
Histidine metabolism	27	115	6.458731e-03	ko00340
TNF signaling pathway	50	259	6.982528e-03	ko04668

Supplementary Table 20 | Summary of the 17 *snFPITE* genes identified from the *S. nudus* genome.

Gene ID	Description	AA length	AA identity	Exon number
snFPITE1	partial CDS, without start, stop codon	164	52.727	3
snFPITE2	Complete CDS	269	99.582	6
snFPITE3	Complete CDS	268	57.261	6
snFPITE4	partial CDS, without start, stop codon	239	55.187	5
snFPITE5	partial CDS, without start codon	164	52.727	3
snFPITE6	partial CDS, without start, stop codon	164	52.121	3
snFPITE7	Complete CDS	258	53.556	5
snFPITE8	partial CDS, without start, stop codon	240	57.438	5
snFPITE9	partial CDS, without stop codon	281	51.867	6
snFPITE10	partial CDS, without start, stop codon	164	50.91%	3
snFPITE11	partial CDS, without start, stop codon	164	50.91%	3
snFPITE12	partial CDS, without start, stop codon	178	50.28%	4
snFPITE13	Complete CDS	277	55.187	6
snFPITE14	partial CDS, without start, stop codon	104	78.846	2
snFPITE15	Complete CDS	267	51.037	6
snFPITE-p1	With premature stop codon	240	/	5
snFPITE-p2	With premature stop codon	166	/	3

Note: aa identity was calculated by comparing with the sequence of snFPITE-c1

Supplementary Table 21 | Protein sequence alignment between full-length transcripts and *snFPITE* genes identified by mass spectroscopy and molecular cloning.

Query id	Query length	Subject id	Subject length	% of identity	Alignment length
snFPITE-n2(A)	238	2k3k0.5to1kb_part0_c39_f1p4_863	863	84.937	239
snFPITE-n2(A)	238	1k2k0.5to1kb_part0_c13_f4p2_880	880	84.937	239
snFPITE-n1	239	2k3k0.5to1kb_part0_c39_f1p4_863	863	87.448	239
snFPITE-n1	239	1k2k0.5to1kb_part0_c13_f4p2_880	880	87.448	239
snFPITE-n1	239	1k2k0.5to1kb_part0_c123_f1p0_858	858	82.427	239
snFPITE-c3	237	2k3k0.5to1kb_part0_c5_f3p10_863	863	81.498	227
snFPITE-c3	237	1k2k0.5to1kb_part0_c0_f12p17_862	862	81.498	227
snFPITE-c3	237	5k10k0.5to1kb_part0_c5_f2p0_862	862	81.057	227
snFPITE-c3	237	1k2k1to1.5kb_part0_c65302_f1p24_1247	1247	81.498	227
snFPITE-c3	237	1k2k1to1.5kb_part0_c861_f7p19_1444	1444	81.498	227
snFPITE-c3	237	3k6k3.5to4kb_part0_c2516_f2p0_3884	3884	80.617	227
snFPITE-c4	232	1k2k0.5to1kb_part0_c14_f4p7_884	884	91.379	232
snFPITE-c4	232	2k3k0.5to1kb_part0_c49_f1p2_880	880	91.379	232
snFPITE-c4	232	5k10k0.5to1kb_part0_c32_f1p0_882	882	88.696	230
snFPITE-c5	232	2k3k0.5to1kb_part0_c49_f1p2_880	880	91.379	232
snFPITE-c5	232	1k2k0.5to1kb_part0_c14_f4p7_884	884	91.379	232
snFPITE-c5	232	5k10k0.5to1kb_part0_c32_f1p0_882	882	89.13	230
snFPITE-c6	242	1k2k0.5to1kb_part0_c24_f2p4_913	913	95.455	242
snFPITE-c7	242	1k2k0.5to1kb_part0_c24_f2p4_913	913	95.041	242
snFPITE-c9	238	2k3k0.5to1kb_part0_c5_f3p10_863	863	91.597	238
snFPITE-c9	238	1k2k0.5to1kb_part0_c0_f12p17_862	862	91.597	238
snFPITE-c9	238	5k10k0.5to1kb_part0_c5_f2p0_862	862	91.176	238
snFPITE-c9	238	1k2k1to1.5kb_part0_c65302_f1p24_1247	1247	91.597	238
snFPITE-c9	238	1k2k1to1.5kb_part0_c861_f7p19_1444	1444	91.597	238
snFPITE-c9	238	3k6k3.5to4kb_part0_c2516_f2p0_3884	3884	90.756	238
snFPITE-c10	239	2k3k0.5to1kb_part0_c39_f1p4_863	863	87.866	239
snFPITE-c10	239	1k2k0.5to1kb_part0_c13_f4p2_880	880	87.866	239
snFPITE-c10	239	1k2k0.5to1kb_part0_c123_f1p0_858	858	81.59	239

Supplementary Table 22 | Nucleotide sequence alignment between full-length transcripts and *snFPITE* genes identified by mass spectroscopy and cloning methods.

Query id	Query length (bp)	Subject ID	Subject length (bp)	% identity	Alignment length (bp)
snFPITE-n1	720	2k3k0.5to1kb_part0_c39_f1p4_863	863	87.639	720
snFPITE-n1	720	1k2k0.5to1kb_part0_c13_f4p2_880	880	87.639	720
snFPITE-n1	720	1k2k0.5to1kb_part0_c123_f1p0_858	858	85.635	717
snFPITE-c3	714	2k3k0.5to1kb_part0_c5_f3p10_863	863	90.336	714
snFPITE-c3	714	1k2k0.5to1kb_part0_c0_f12p17_862	862	90.336	714
snFPITE-c3	714	5k10k0.5to1kb_part0_c5_f2p0_862	862	90.196	714
snFPITE-c3	714	1k2k1to1.5kb_part0_c65302_f1p24_1247	1247	90.196	714
snFPITE-c3	714	1k2k1to1.5kb_part0_c861_f7p19_1444	1444	90.196	714
snFPITE-c3	714	3k6k3.5to4kb_part0_c2516_f2p0_3884	3884	89.776	714
snFPITE-c4	696	1k2k0.5to1kb_part0_c14_f4p7_884	884	92.098	696
snFPITE-c4	696	5k10k0.5to1kb_part0_c32_f1p0_882	882	91.954	696
snFPITE-c4	696	2k3k0.5to1kb_part0_c49_f1p2_880	880	91.81	696
snFPITE-c4	696	1k2k0.5to1kb_part0_c67_f1p1_860	860	85.441	680
snFPITE-c5	696	1k2k0.5to1kb_part0_c14_f4p7_884	884	91.81	696
snFPITE-c5	696	5k10k0.5to1kb_part0_c32_f1p0_882	882	91.667	696
snFPITE-c5	696	2k3k0.5to1kb_part0_c49_f1p2_880	880	91.523	696
snFPITE-c5	696	1k2k0.5to1kb_part0_c67_f1p1_860	860	85.147	680
snFPITE-c6	729	1k2k0.5to1kb_part0_c24_f2p4_913	913	92.72	728
snFPITE-c7	726	1k2k0.5to1kb_part0_c24_f2p4_913	913	92.562	726
snFPITE-c9	714	2k3k0.5to1kb_part0_c5_f3p10_863	863	89.496	714
snFPITE-c9	714	1k2k0.5to1kb_part0_c0_f12p17_862	862	89.496	714
snFPITE-c9	714	5k10k0.5to1kb_part0_c5_f2p0_862	862	89.356	714
snFPITE-c9	714	1k2k1to1.5kb_part0_c65302_f1p24_1247	1247	89.356	714
snFPITE-c9	714	1k2k1to1.5kb_part0_c861_f7p19_1444	1444	89.356	714
snFPITE-c9	714	3k6k3.5to4kb_part0_c2516_f2p0_3884	3884	89.076	714
snFPITE-c10	720	2k3k0.5to1kb_part0_c39_f1p4_863	863	87.917	720
snFPITE-c10	720	1k2k0.5to1kb_part0_c13_f4p2_880	880	87.917	720
snFPITE-c10	720	1k2k0.5to1kb_part0_c123_f1p0_858	858	85.216	717

Supplementary Table 23 | Alignment between 14 full-length transcripts and 17 *snFPITE* genes identified from the assembled *S. nudus* genome.

Query id	Query length (bp)	Subject id	Subject length (bp)	% identity	Alignment length (bp)
snFPITE 4	717	2k3k0.5to1kb_part0_c39_f1p4_863	863	99.861	717
snFPITE 4	717	1k2k0.5to1kb_part0_c13_f4p2_880	880	99.861	717
snFPITE 4	717	1k2k0.5to1kb_part0_c123_f1p0_858	858	88.703	717
snFPITE 3	807	2k3k0.5to1kb_part0_c39_f1p4_863	863	94.052	807
snFPITE 3	807	1k2k0.5to1kb_part0_c13_f4p2_880	880	94.052	807
snFPITE 3	807	1k2k0.5to1kb_part0_c123_f1p0_858	858	91.542	804
snFPITE 7	777	2k3k0.5to1kb_part0_c5_f3p10_863	863	92.782	762
snFPITE 7	777	1k2k0.5to1kb_part0_c0_f12p17_862	862	92.782	762
snFPITE 7	777	5k10k0.5to1kb_part0_c5_f2p0_862	862	92.651	762
snFPITE 7	777	1k2k1to1.5kb_part0_c65302_f1p24_1247	1247	92.651	762
snFPITE 7	777	1k2k1to1.5kb_part0_c861_f7p19_1444	1444	92.651	762
snFPITE 7	777	3k6k3.5to4kb_part0_c2516_f2p0_3884	3884	92.388	762
snFPITE 9	843	5k10k0.5to1kb_part0_c5_f2p0_862	862	74.241	823
snFPITE 9	843	2k3k0.5to1kb_part0_c5_f3p10_863	863	74.272	824
snFPITE 9	843	1k2k1to1.5kb_part0_c65302_f1p24_1247	1247	74.272	824
snFPITE 9	843	1k2k0.5to1kb_part0_c0_f12p17_862	862	74.272	824
snFPITE 9	843	1k2k1to1.5kb_part0_c861_f7p19_1444	1444	74.272	824
snFPITE 9	843	3k6k3.5to4kb_part0_c2516_f2p0_3884	3884	74.029	824

Supplementary methods for Mass Spectormetry of snFPITE-1 and -2

1. Digestion

i. In-solution digestion

50 μg sample was added in UA buffer, DTT and iodoacetamide were added to

reduce and block the cysteine residues. Then trypsin was added respectively. The mixtures were all incubated at 37°C for 16-18 h.

ii. Filter-aided sample preparation (FASP Digestion)

200 μg of proteins for each sample were incorporated into 30 μl SDT buffer (4% SDS, 100 mM DTT, 150 mM Tris-HCl pH 8.0). The detergent, DTT and other low-molecular-weight components were removed using UA buffer (8 M Urea, 150 mM Tris-HCl pH 8.0) by repeated ultrafiltration (Microcon units, 10 kD). Then 100 μl iodoacetamide (100 mM IAA in UA buffer) was added to block reduced cysteine residues and the samples were incubated for 30 min in darkness. The filters were washed with 100 μl UA buffer three times and then 100 μl 25mM NH_4HCO_3 buffer twice. Finally, the protein suspensions were digested with 4 μg trypsin (Promega) in 40 μl 25mM NH_4HCO_3 buffer overnight at 37 °C, and the resulting peptides were collected as a filtrate. The peptides of each sample were desalted on C18 Cartridges (Empore™ SPE Cartridges C18 (standard density), bed I.D. 7 mm, volume 3 ml, Sigma), concentrated by vacuum centrifugation and reconstituted in 40 µl of 0.1% (v/v) formic acid. The peptide content was estimated by UV light spectral density at 280 nm using an extinctions coefficient of 1.1 of 0.1% (g/l) solution that was calculated on the basis of the frequency of tryptophan and tyrosine in vertebrate proteins.

2. Mass Spectrometry

HPLC

Each fraction was injected for nanoLC-MS/MS analysis. The peptide mixture was loaded onto a reverse phase trap column（Thermo Scientific Acclaim PepMap100, 100μm*2cm, nanoViper C18）connected to the C18-reversed phase analytical column (Thermo Scientific Easy Column, 10 cm long, 75 μm inner diameter, 3μm resin) in buffer A (0.1% Formic acid) and separated with a linear gradient of buffer B (84% acetonitrile and 0.1% Formic acid) at a flow rate of 300 nl/min controlled by IntelliFlow technology. The linear gradient was determined by the project proposal:

i. 0.5 hour gradient：5-35% buffer B for 22 min, 35-100% buffer B for 5 min, hold in 100% buffer B for 3 min.

ii. 1 hour gradient：0-60% buffer B for 50 min, 60-90% buffer B for 4 min, hold in 90%

buffer B for 6 min.

iii. 2 hours gradient：0-40% buffer B for 110 min, 40-90% buffer B for 5 min, hold in 90% buffer B for 5 min.

iv. 4 hours gradient：0-40% buffer B for 225 min, 40-100% buffer B for 5 min, hold in 100% buffer B for 10 min.

LC-MS/MS Analysis

LC-MS/MS analysis was performed on a Q Exactive mass spectrometer (Thermo Scientific) that was coupled to Easy nLC (Proxeon Biosystems, now Thermo Fisher Scientific) for 30 min. The mass spectrometer was operated in positive ion mode. MS data was acquired using a data-dependent top20 method dynamically choosing the most abundant precursor ions from the survey scan (300–1800 m/z) for HCD fragmentation. Automatic gain control (AGC) target was set to 1e6, maximum inject time to 50 ms， and number of scan ranges to 1. Dynamic exclusion duration was 30.0 s. Survey scans were acquired at a resolution of 70,000 at m/z 100 and resolution for HCD spectra was set to 17,500 at m/z 100, Automatic gain control (AGC) target was set to 1e5,isolation width was 1.5 m/z, microscans to 1, and maximum inject time to 50 ms. Normalized collision energy was 27 eV and the underfill ratio, which specifies the minimum percentage of the target value likely to be reached at maximum fill time, was defined as 0.1%. The instrument was run with peptide recognition mode enabled.

3. Data Analysis

MS/MS spectra were searched using MASCOT engine (Matrix Science, London, UK; version 2.2) against a nonredundant International Protein Index arabidopsis sequence database v3.85 (released at September 2011; 39679 sequences) from the European Bioinformatics Institute (http://www.ebi.ac.uk/). For protein identification, the following options were used. Peptide mass tolerance=20 ppm, MS/MS tolerance=0.1 Da, Enzyme=Trypsin, Missed cleavage=2, Fixed modification: Carbamidomethyl (C), Variable modification：Oxidation(M)

Project description:

In this project, we purified proteins snFPITE-n1 and snFPITE-n2 from Sipunculus nudus, digested the proteins and determined the sequences of the proteins by Mass spec analysis. We have a database of the sequences of snFPITE proteins from Sipunculus nudus and we used the Mass spec results to search for the protein sequences from this database.
