# Supplementary material for: A facultative plasminogen-independent thrombolytic enzyme from Sipunculus nudus
Source: Nat Commun. 2025 Apr 24;16:3852. doi: 10.1038/s41467-025-58915-y (PMC12022309; doi:10.1038/s41467-025-58915-y)
Supplement: Supplementary file 2 — Description for the Supplementary Data [file 41467_2025_58915_MOESM2_ESM.pdf]

Description of Additional Supplementary Files

File Name: Supplementary Data 1

Description: MS data of snFPITE-n1.

File Name: Supplementary Data 2

Description: MS data of snFPITE-n2.

File Name: Supplementary Data 3

Description: Significantly enriched KEGG pathway of *S. nudus* genes in unique gene families.

File Name: Supplementary Data 4

Description: Nucleotide sequence alignment between full-length transcripts and snFPITE genes identified by mass spectroscopy and cloning methods.

File Name: Supplementary Data 5

Description: Alignment between 14 full-length transcripts and 17 snFPITE genes identified from the assembled *S. nudus* genome.
